# Interactive balance training integrating sensor-based visual feedback of movement performance: a pilot study in older adults

**DOI:** 10.1186/1743-0003-11-164

**Published:** 2014-12-13

**Authors:** Michael Schwenk, Gurtej S Grewal, Bahareh Honarvar, Stefanie Schwenk, Jane Mohler, Dharma S Khalsa, Bijan Najafi

**Affiliations:** Interdisciplinary Consortium on Advanced Motion Performance (iCAMP), Department of Surgery, College of Medicine, University of Arizona, Tucson, AZ USA; Arizona Center on Aging, University of Arizona, Tucson, AZ USA; Alzheimer’s Research and Prevention Foundation, Tucson, AZ USA

**Keywords:** Wearable sensors, Exercise, Exergame, Interactive, Balance, Postural control, Older adults, Fall risk

## Abstract

**Background:**

Wearable sensor technology can accurately measure body motion and provide incentive feedback during exercising. The aim of this pilot study was to evaluate the effectiveness and user experience of a balance training program in older adults integrating data from wearable sensors into a human-computer interface designed for interactive training.

**Methods:**

Senior living community residents (mean age 84.6) with confirmed fall risk were randomized to an intervention (IG, n = 17) or control group (CG, n = 16). The IG underwent 4 weeks (twice a week) of balance training including weight shifting and virtual obstacle crossing tasks with visual/auditory real-time joint movement feedback using wearable sensors. The CG received no intervention. Outcome measures included changes in center of mass (CoM) sway, ankle and hip joint sway measured during eyes open (EO) and eyes closed (EC) balance test at baseline and post-intervention. Ankle-hip postural coordination was quantified by a reciprocal compensatory index (RCI). Physical performance was quantified by the Alternate-Step-Test (AST), Timed-up-and-go (TUG), and gait assessment. User experience was measured by a standardized questionnaire.

**Results:**

After the intervention sway of CoM, hip, and ankle were reduced in the IG compared to the CG during both EO and EC condition (p = .007-.042). Improvement was obtained for AST (p = .037), TUG (p = .024), fast gait speed (p = . 010), but not normal gait speed (p = .264). Effect sizes were moderate for all outcomes. RCI did not change significantly. Users expressed a positive training experience including fun, safety, and helpfulness of sensor-feedback.

**Conclusions:**

Results of this proof-of-concept study suggest that older adults at risk of falling can benefit from the balance training program. Study findings may help to inform future exercise interventions integrating wearable sensors for guided game-based training in home- and community environments. Future studies should evaluate the added value of the proposed sensor-based training paradigm compared to traditional balance training programs and commercial exergames.

**Trial registration:**

http://www.clinicaltrials.govNCT02043834.

**Electronic supplementary material:**

The online version of this article (doi:10.1186/1743-0003-11-164) contains supplementary material, which is available to authorized users.

## Introduction

Aging has a detrimental effect on postural control as a consequence of general age-related deterioration of sensory and neuromuscular control mechanisms and/or specific pathologies [1]. Impaired postural control can have serious consequences regarding physical functioning and is a predictor for falls in older adults [2].

Balance training is considered to be an important aspect of a fall prevention program [3]. However, a drawback of conventional exercise programs developed to improve balance is low adherence, particularly in unsupervised home/community settings [4]. Emerging applications incorporate wearable sensors to facilitate the implementation of home-based rehabilitation interventions [5]. Sensor-based training programs may have several advantages compared to conventional exercises including interactive environments responsive to the user’s action, feedback for motor skill acquisition, incorporation of gaming features, and targeted intervention incorporating guided home exercising without the expense of a personal trainer [5–7].

Wearable sensor-based systems for the purpose of balance training in frail older adults may offer advantages when compared to commercial exergame systems based on force platforms or camera systems. Commercial videogames using off-the-shelf camera systems (i.e., Microsoft Kinect) may have limited accuracy to measure balance performance [8, 9]. It has been recently suggested that incorporating wearable sensors into commercial videogames could improve accuracy of balance assessment [10]. Unlike camera-based systems, wearable sensors do not require a continuous unobstructed sightline. Thus, exercises can be safely conducted while standing behind a chair, enabling subjects to grab the backrest if needed. Commercial force platforms (i.e., Nintendo Wii), which restrict the base of support, may increase risk of falls during training [11, 12] thus limiting the usability in frail older adults. Sensor-based systems do not require standing on force platforms, allowing subjects to exercise on the ground and to select a natural stance without regard to force platform position. In summary, assuring safety during unsupervised exergaming is often challenging when using commercial systems, which were not developed for frail older adults who are at high fall risk. These observations demonstrate the importance for designing targeted technological applications for balance training in frail older adults. However, current studies have focused predominately on healthy older individuals [13–17] and have used off-the-shelf systems that were more appropriate for balance training in high functioning subjects (i.e., Wii Fit package [15, 17], Wii sport package [18], and Dancetown dance mat [16]).

Training of postural control requires appropriate tracking and feedback of performance [19]. Wearable sensor technology can accurately measure postural control [20, 21] and may provide a new avenue for motion feedback during balance training. Incorporation of wearable sensors into balance training has been repeatedly suggested in review articles [6, 7]; however, to our knowledge, this approach has not yet been evaluated in older adults.

The current research focuses on the evaluation of a new wearable sensor-based exercise training regimen specifically developed to improve balance [22]. The exercise system integrates data from wearable sensors into a human-computer interface designed for game-based training. A key feature of the system is its ability to measure lower extremity three-dimensional movement for providing real-time feedback in order to assist and motivate the user during training.

The primary aim of this pilot study was to evaluate the user-friendliness and effectiveness of the new balance exercise regimen based on wearable sensors in older adults living in a senior living community. We hypothesized that 4-weeks of balance training (twice a week, each session 45 min) would result in improved balance performance in our sample of participants. Previous studies in older adults have described balance improvements after a comparable dosage of balance training (Young et al.: 4-weeks, 10 sessions á 20 min [23]; Hu et al.: 15-days, 10 sessions á 60 min [24]), supporting our hypothesis. The second aim was to explore whether the balance training improved functional performance.

## Methods

### Study design

The study was designed as a single-blinded, randomized, controlled intervention trial. Investigators were not aware of group assignment. The trial was registered at http://www.clinicaltrials.gov (NCT02043834). The study was approved by the University of Arizona Institutional Review Committee (approval no. 13–0538).

### Study population

Individuals were recruited from a senior living community (Villa Hermosa, Tucson, AZ). Recruitment started in November 2013 and post-intervention assessment was completed in May 2014. Inclusion criteria were (1) age 65 and older, (2) presence of fall risk (Timed-up-and-go ≥12 sec [25]), (3) ability to walk without an assistive device for a minimum of 10 meters, and (4) written informed consent. Exclusion criteria included (1) cognitive impairment (Mini-Mental State Examination ≤ 23 points [26]), (2) neurological disorders, (3) severe visual impairment, (4) uncontrolled/terminal cardiovascular, metabolic, or psychiatric disorders, and (5) participation in previous balance training. Exclusion criteria 2–5 were obtained by self-report.

**Figure 1:**
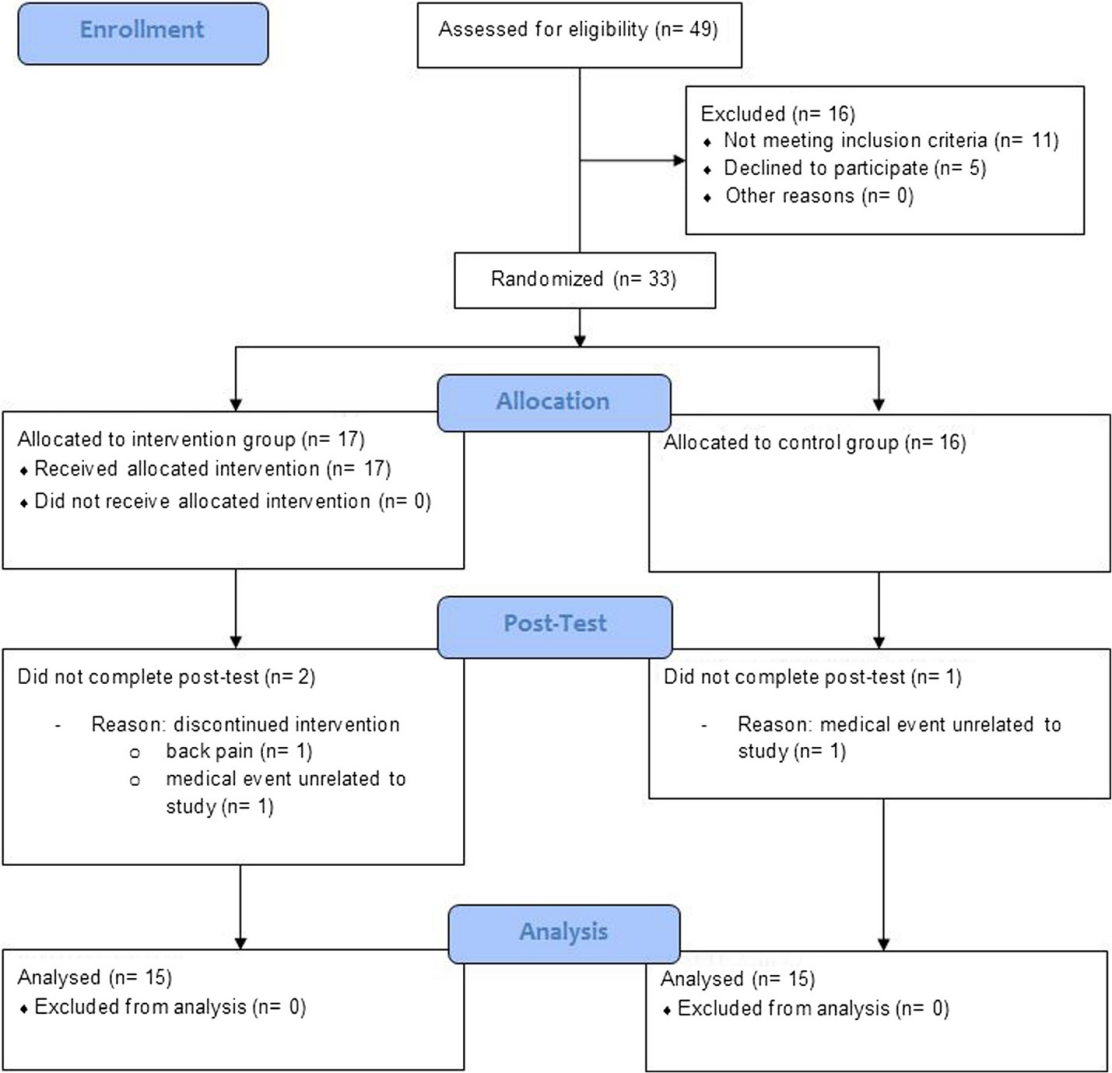
**CONSORT flow diagram of progress through the phases of screening, enrolment, allocation, post-testing, and data analysis.**

Subjects meeting the inclusion criteria were randomly assigned to the intervention group (IG), or the control group (CG), using the urn design [27] (numbered containers). The sequence was concealed until group assignment (after baseline measurement) was completed. A person unrelated to the study performed the randomization procedure. The progress through the phases of screening, enrolment, allocation, post-testing, and data analysis is illustrated in Figure 1.

### Intervention

#### Exercise training technology

**Figure 2:**
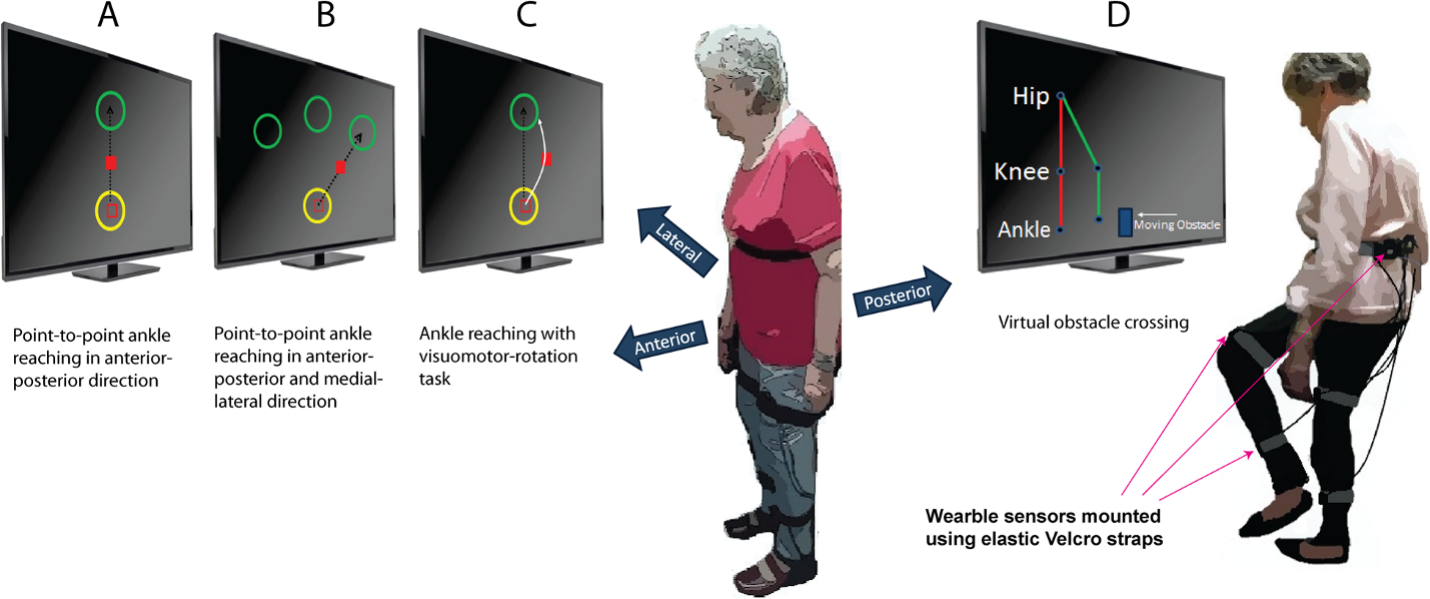
**An illustration of the interactive balance training program. A**: The ankle reaching task involves moving a red dot from a start circle (yellow) to a target circle (green) in a straight line. **B**: The ankle reaching task is conducted in anterior-posterior and medial-lateral direction. **C**: The trajectory of the cursor is rotated by an angle of 20°. The participant needs to observe this change in trajectory during the reaching task and compensate by adjusting ankle/hip coordination. **D**: The participant is challenged to cross virtual obstacles appearing on the screen. Lower extremity feedback is provided by wearable sensors.

We used technology specifically developed for assessment and training of postural control, as described previously [21, 22, 28]. It included a 24-inch computer screen, an interactive game-based virtual interface designed in MatLab® 2007a and Psych toolbox V2.54, and 5 wearable inertial sensors (LegSys™, BioSensics LLC, MA, USA) providing tri-axial accelerometer, gyroscope and magnetometer data along with quaternion parameters; ideal for estimation of three dimensional joint angles and position [28]. The data were acquired and transmitted at a frequency of 100 Hz; used for real-time visual feedback in virtual environment. In order to acquire motion quality kinematic data, the sensors were mounted on different body segments including each shank, thigh and lower back using elastic straps (Figure 2).

#### Training procedure

The equipment was set up in a quiet room in the Senior Living Community. During training, the participant stood in front of a computer screen which was positioned at eye-level. For safety purposes, a chair was placed in front of the participant to provide support, if needed. A study coordinator explained the balance tasks to the participant during the first session. During subsequent sessions, participants conducted training using sensor-based feedback only. The study coordinator remained with the participant during training sessions to guarantee safety.

#### Training protocol

Participants in the IG attended training sessions twice a week, for 4 weeks. Each session lasted approximately 45 min and included: 1) ankle point-to-point reaching tasks; and 2) virtual obstacle crossing tasks, as described in detail below. Frequency and total number of sessions was determined based on our previous pilot study in patients with diabetic peripheral neuropathy [29], which found a significant reduction in center of mass (CoM) sway using the identical training protocol. The goal of the training was to teach the participant to control the movement of the lower extremity and CoM during leaning and weight shifting tasks. Exercises were chosen to improve movement skills thought to underlie postural balance [3]. The game-based interface was designed to be intuitive and easy to play, whilst avoiding distracting, complex animations (Figure 2 A-D). The animation was either a red square providing feedback about ankle movement during the ankle reaching task or a stick figure avatar of the lower limb during the virtual obstacle crossing task (described in detail below). The simplistic design of the graphical user interface was chosen to allow the participant to focus on the exercise tasks and to better perceive motor error (difference between the actual motor output and the desired motor output) during exercise, which is considered to be one of the major sources of motor learning [30]. No other graphical effects were included in the interface as they may distract older adults [11].

#### Ankle point-to-point reaching task

This task has been implemented previously [22]. The aim of the ankle reaching task was to help participants learn weight shifting, and coordinate proximal (hip) and distal (ankle) joints. The exercise also encourages forward/backward/sideward leaning (Figure 2A-C) and partial weight transfer that has been found to improve postural balance [31].

The reaching task was performed using data from shank mounted sensor. More specifically, the kinematic data related to the rotation around ankle joint was translated into linear movement on computer screen. Two circles appeared sequentially on the screen; a start circle in yellow followed by a target circle in green (Figure 2A). After a visual start signal participants navigated a red cursor from the start circle to the target circle by rotating the ankle joint. The task was repeated from the target to the start circle to complete one cycle of ankle reaching task. Participants were expected to move rapidly (<1 second) and accurately (navigate cursor in middle of circle) from circle to circle. Upon reaching a circle in < 1 second, participants were awarded for correct task execution using visual (circle explodes) and audio (positive sound) feedback. If moving too slowly (>1 second), the participants received visual feedback about the incorrect task execution (circle changed to blue colour).

During the ankle reaching task the participant stood upright and was instructed to move the hips in anterior-posterior direction in order to generate ankle dorsiflexion/plantar-flexion (for moving the cursor forward and backward between circles, Figure 2A). Similarly, medial-lateral hip movement navigated the cursor sideward (Figure 2B).

Each training session consisted of a total of 6 blocks each with 20 cycles of ankle reaching tasks. Training blocks 1 and 2 focused on ankle reaching in anterior-posterior direction (Figure 2A). Block 3 and 4 included ankle reaching in combined anterior-posterior and medial-lateral direction (Figure 2B). In order to increase the challenge, the final two blocks (5 and 6) were conducted with visuomotor rotation [32]. The trajectory of the cursor representing the ankle joint motion was rotated by a 20° angle on the screen (Figure 2C). Participants were expected to observe this change in trajectory during the reaching task and compensate by adjusting ankle coordination in order to move the cursor to the target location in a point-to-point straight line. The visuomotor rotation task aimed to improve participant’s postural adaptation strategies in order to achieve a more permanent postural calibration, as described previously [33]. One minute break was given between successive blocks in order avoid fatigue. All participants started with the ankle-reaching task in the anterior-posterior direction. When participants were able to conduct this task without making a significant amount of errors they progressed to more advanced medial-lateral ankle reaching task and visual rotation task. In this pilot study progression was performed based on the judgement of the supervisor.

#### Virtual obstacle crossing task

Participants were challenged to cross a series of virtual obstacles (boulders) approaching on the screen from right to left (Figure 2D). The participant was standing in front of the computer screen and raised the leg in the sagittal plane (hip/knee flexion, ankle dorsiflexion) in order to cross an obstacle. Kinematic data from all the 5 inertial sensors provided real-time feedback using a simple stick figure avatar representing the participant’s lower extremities on the screen. The figure replicated lower extremity movement including lifting of designated foot to appropriate height in order to cross the obstacle. The stick figure avatar was shown in a 2D side perspective on the screen and the virtual obstacle appeared at the right end of the monitor and moved towards the avatar (Figure 2 D). Previous studies suggest that that the chosen 2D side perspective is superior to 3D behind and “ego” perspectives for perceiving visual feedback during virtual obstacle crossing [34]. The feedback on the distance between avatar and next obstacle can be perceived most clearly with the side perspective [34].

Each training session included three series of obstacle crossing with 15 repetitions each, with progressing obstacle height (10%, 15%, and 20% of leg length). Obstacle height was individually increased based on the performance of the participant. Participants received audio and visual feedback at the end of each obstacle-crossing trial which indicated whether they successfully crossed the obstacle or not. To cognitively challenge the participants, they were instructed to alternate between right and left foot during an obstacle crossing sequence. If participants forgot the sequence of foot lifting, the foot on the screen moved downward instead of lifting to notify the wrong sequence. In this case, participants needed to repeat the trial with the correct foot. Participants were stationary during obstacle crossing exercise and standing behind a chair, to receive support if needed.

### Measurements

Outcome measurements were performed at baseline and post-intervention using validated tests.

#### Clinical characteristics

Clinical characteristics including comorbidity (number of diagnoses), prescriptions (number), BMI, functional status (Barthel Index [35]), cognitive status (MMSE), depressive signs (Center for Epidemiologic Studies Depression Scale [36]), health–related quality of life (Short-Form Health Survey [37]), fear of falling (Short Falls Efficacy Scale International, Short-FES-I, [34]), pain (Visual Analogue Scale) and falls (past year) were documented by standardized interviewer-administered assessment.

#### Motor performance

**Primary outcome measure** Balance: Balance was assessed using wearable technology (BalanSens™, BioSensics, MA, USA) consisting of three inertial sensors attached to right and left shank and lower back. Balance was measured during 30-second standing with feet close together (but not touching) with eyes open (EO), and eyes closed (EC). The CoM sway was calculated by a validated algorithm [21]. The CoM sway area (cm2) during EO stance was defined as the primary outcome measure.

Sample size was calculated for the primary outcome measure (training-related reduction in CoM sway area during EO stance) using results of our previous study in patients with diabetic peripheral neuropathy [29]. In this study CoM sway was reduced from 3.1 ± 2.92 cm^2^ to 1.25 ± 1.32 cm^2^ after 4 weeks of training. Assuming an effect size of d = 0.73, power of 80%, significance level of .05, and drop-out-rate of 10%, a sample size of 30 (15 per group) was needed to verify a significant effect.

**Secondary outcome measures** Balance: Beside CoM sway area (primary outcome measure, described above), the anterior-posterior (AP, cm) and medial-lateral (ML, cm) CoM sway components were calculated using validated algorithms [21]. Further, the hip sway (deg2) and the ankle sway (deg2) were calculated [21]. Additionally, we explored postural coordination strategy (reduction in CoM sway through coordination of hip and ankle motion) quantified by a validated reciprocal compensatory index (RCI), which has been described by Najafi et al. in detail [21]. Briefly, using wearable sensors described above, ankle, hip, and CoM sway were estimated. RCI was then calculated as function of correlation between movement of the hip and ankle joints using the following formula:

$$\text{RCI}=\sqrt{1+\frac{2\text{rk}1k2\;\sqrt{\text{var}\left(\text{sin}\left(\theta a\right)\right),\;\text{var}\left(\text{sin}\left(\theta h\right)\right)\;}}{k1^{2}\text{var}\left(\text{sin}\left(\theta a\right)\right)+k2^{2}\text{var}\left(\text{sin}\left(\theta h\right)\right)}}$$

Where ‘r’ represents the coefficient of correlation between ankle and hip movement, ‘var’ denotes variance, and θa and θh denote, respectively, ankle and hip angles in any given time. K1 and k2 are constants and are estimated using subject's anthropometry data as described in Najafi et al. [21]. As demonstrated by Najafi et al. via simulation, RCI values closer to zero indicate better reciprocal coordination [21]. The reciprocal coordination allows subjects to compensate the proximal segment movement (hip) via anticipation of the distal segment movement (ankle) and vice versa (i.e., negative correlation between hip and ankle movements). RCI values more than 1 represent inappropriate postural control (i.e., positive correlation between hip and ankle movements, leading to increased CoM variations) [21]. In patients with impaired somatosensory feedback (diabetic peripheral neuropathy), reciprocal coordination was lower (RCI: 0.90 ± 0.11 and 0.89 ± 0.14 for AP and ML) compared to healthy control subjects (RCI: 0.70 ± 0.01 and 0.80 ± 0.05) [21]. Additionally, in patients with impaired somatosensory feedback, RCI was increased to 0.95 ± 0.21 and 0.96 ± 0.20, respectively for AP and ML, while eyes closed during balance test [21]. The RCI is a relatively new index of balance performance and, to our knowledge, no validated normative values exist for the general population.

Alternate step test (AST): The AST is a validated stepping task which can predict falls risk [38]. The test involves placing the whole foot onto a step, which is 18-cm high and 40-cm deep, and alternating with the right and left feet for a total of eight repetitions as quickly as possible. The time taken to complete the task is the score.

Instrumented gait analysis: Gait performance was assessed using wearable technology (LegSys™, BioSensics, MA, USA). Participants walked a distance of 10 meters at habitual speed, and as fast as possible. Gait speed and gait variability defined as coefficient of variation [CV] of stride velocity were calculated using a validated algorithm [39].

Timed up and go test (TUG): The TUG is a valid clinical test to quantify mobility performance by timing participants with a stopwatch while rising from an armchair, walking 3 meters, turning, walking back, and sitting down [40].

User experience: User experience was evaluated using a standardized questionnaire originally developed for evaluating the Wii balance board [11]. It consisted of ten 5-level Likert-scale questions (0 = completely disagree to 4 = absolutely agree, 2 = neutral) which were adapted to the sensor technology used in this study.

### Statistical analysis

Unpaired t-tests and Chi-square-tests were used for baseline comparisons according to the scale of the investigated variable. Analysis of covariance (ANCOVA) was used to compare the effect of the intervention on post-intervention outcome parameters while adjusting for baseline values [41]. The primary study endpoint was reduction in CoM sway area during EO stance. All other variables were secondary outcomes. Effect sizes were calculated from ANCOVA as partial eta squared (η_p_^2^). Values ranging from 0.01 to 0.06 indicate small; from 0.06 to 0.25 medium, and above 0.25 large effects [42]. Univariate linear regression analyses were performed to delineate predictive factors of training response for the primary study endpoint (pre- to post- changes in CoM sway area with EO). Variables included baseline parameters including age, gender, ADL-status, comorbidity, cognitive performance, depression, and baseline motor variables (CoM sway, gait speed, AST). Results are given as regression coefficients β and fit of the model is reported by coefficient of determination R^2^.

Associations between pre- to post changes in balance parameters and changes in functional performance (AST, gait speed, TUG) were quantified by Pearson’s correlation. Correlations were considered low (r < 0.2), moderate (r = 0.2-0.5), or high (r > 0.5). A p-value ≤ 0.05 was considered to be statistically significant. Statistical analysis was performed using SPSS statistics 17.0 (IBM, Armonk, NY, USA).

## Results

Thirty-three subjects were recruited into the study (Figure 1). Three participants (9.1%; 2 IG, 1 CG) dropped out during the intervention period. Reasons for drop outs were back pain after the first training session (n = 1; IG), and hospitalization due to acute medical events unrelated to the study (n = 2; 1 IG, 1CG). Training adherence was excellent: 14 IG participants (93.3%) completed the 8 training sessions; one participant (6.7%) conducted 6 training sessions only (75%) due to relocation to another residence. All participants adjusted to the interactive training program during the first training session within 10–15 minutes. Training was safe despite the participant’s advanced ages and functional impairment. No training-related adverse events occurred.

The participants, average age was 84.6 ± 6.8 years, and MMSE averaged 28.4 ± 1.6 points. Habitual gait speed averaged 0.80 ± 0.17 meters per second, representing the speed of frail older adults [43]. TUG averaged 17.4 seconds, indicating low functional performance and increased risk of falling [25]. Fear of falling was moderate (Short-FES-I = 9–13 points) in eleven participants (33.3%), and high (Short-FES-I ≥ 18 points) in 18 (54.5%) participants. Eighteen participants (54.5%) reported 1 or more falls in the last year. No differences between IG and CG were found for any baseline variable (Table 1). Also, no differences were found between dropout-adjusted groups (p = .148-1.00) suggesting no systematic bias due to dropouts.

**Table 1:** **Baseline characteristics of study participants**

Characteristic	Intervention (n = 17)	Control (n = 16)	P-value
Age, years	84.3 ± 7.3	84.9 ± 6.6	.792
Women, number	10 (58.8)	11 (68.8)	.554
BMI, kg/m^2^	26.3 ± 6.1	27.4 ± 6.6	.631
Mini Mental State Examination, score	28.7 ± 1.4	28.0 ± 1.7	.210
Barthel Activities of Daily Living, score	92.7 ± 5.0	92.2 ± 8.9	.856
CES-D scale, score	8.3 ± 6.6	8.5 ± 6.8	.931
SF-12, Physical Component, score	37.7 ± 10.0	35.1 ± 5.9	.369
SF-12, Mental Component, score	54.1 ± 8.7	54.6 ± 9.6	.862
Short Fall Efficacy Scale, score	13.2 ± 4.0	14.9 ± 5.1	.276
Diagnoses, number	3.1 ± 1.4	3.1 ± 1.0	.987
Prescriptions, number	4.4 ± 2.3	5.0 ± 2.9	.501
Visual Analogue Pain Scale (0–10), score	2.8 ± 2.5	2.9 ± 2.7	.955
History of falls in the last year, number of participants	9 (53)	9 (56)	.849
Timed up and go, sec	17.1 ± 4.9	17.8 ± 4.8	.659
Gait speed, habitual, m/sec	0.83 ± 0.15	0.77 ± 0.20	.324

Data are mean ± standard deviation or number (%); P- values are given for difference between the intervention and control group; SF, Short Form Health Survey; CES-D, Center for Epidemiological Studies Depression Scale.

### Effect of the intervention on balance and functional performance

Results of baseline and post-test balance assessment are reported in Table 2. With EO, sway of CoM (area, ML, AP), hip and ankle was reduced in the IG compared to CG after the intervention (p = .007-.030). Effect sizes were moderate with highest effects for CoM sway area (η_p_^2^ = .239) and lowest for ankle sway (η_p_^2^ = .162). With EC, sway of CoM (area, ML), hip, and ankle was reduced in the IG compared to the CG (p = .010-.042). Effect sizes were moderate with highest effects for hip sway (η_p_^2^ = .222) and lowest for CoM sway area (η_p_^2^ = .144). AP CoM sway did not change with EC (p = .142). Postural coordination (RCI) showed a trend of improvement with EO in AP (p = .051) but not in ML direction (p = .888). RCI did not change with EC (p = .521-.608).

**Table 2:** **Effects of the interactive balance training on postural balance parameters**

Parameters	Control group			Intervention group			P value^b^	Effect size^c^
	Baseline n = 15	Post-test n = 15	% change^a^	Baseline n = 15	Post-test n = 15	% change^a^		
**Eyes open**								
CoM sway, area, cm^2^	2.51 ± 1.86	2.62 ± 1.66	-4.4	3.03 ± 2.29	1.45 ± 1.01	52.2	.007	.239
CoM sway, ML, cm	1.86 ± 0.86	1.82 ± 0.56	2.2	2.07 ± 0.83	1.43 ± 0.38	30.9	.016	.196
CoM sway, AP, cm	1.22 ± 0.55	1.33 ± 0.48	-9.0	1.30 ± 0.68	0.93 ± 0.50	28.5	.015	.201
Hip sway, deg^2^	1.20 ± 0.93	1.32 ± 0.67	-10.0	1.50 ± 1.07	0.92 ± 0.65	38.7	.011	.214
Ankle sway, deg^2^	1.21 ± 0.94	1.37 ± 1.16	-13.2	1.12 ± 0.79	0.64 ± 0.35	42.9	.030	.162
RCI, AP	0.57 ± 0.22	0.60 ± 0.16	-5.3	0.57 ± 0.12	0.51 ± 0.08	10.5	.051	.134
RCI, ML	0.87 ± 0.05	0.90 ± 0.10	-3.5	0.88 ± 0.13	0.90 ± 0.07	-2.3	.888	.001
**Eyes closed**								
CoM sway, area, cm^2^	5.74 ± 5.22	5.52 ± 6.97	3.8	5.72 ± 4.37	2.36 ± 2.25	58.7	.042	.144
CoM sway, ML, cm	2.53 ± 1.06	2.46 ± 1.39	2.8	2.80 ± 1.28	1.73 ± 0.70	38.2	.012	.214
CoM sway, AP, cm	1.93 ± 1.15	1.71 ± 1.16	11.4	1.76 ± 0.96	1.19 ± 0.71	32.4	.142	.078
Hip sway, deg^2^	2.30 ± 2.11	3.20 ± 5.00	-39.1	3.03 ± 2.37	1.12 ± 0.82	63.0	.010	.222
Ankle sway, deg^2^	2.69 ± 2.54	2.09 ± 1.86	22.3	2.40 ± 1.91	1.02 ± 0.89	57.5	.026	.170
RCI, AP	0.57 ± 0.14	0.58 ± 0.13	-1.8	0.56 ± 0.12	0.55 ± 0.14	1.8	.608	.010
RCI, ML	0.85 ± 0.09	0.88 ± 0.07	-3.5	0.91 ± 0.10	0.89 ± 0.08	2.2	.876	.001

Data presented as mean ± standard deviation; ^a^positive scores indicate improvement; ^b^P-values from ANCOVA comparing the effect of the intervention on post-test outcome parameters adjusting for baseline values; ^c^Effect size eta squared from ANCOVA; CoM, center of mass; ML, medial-lateral; AP, anterior-posterior; RCI, reciprocal compensatory index.

Improvements were obtained for AST (p = .037), TUG (p = .024), and gait speed during fast walking (p=. 010) but not during normal walking condition (p = .264). Gait variability did not change under either condition (p = .902-.951) (Table 3).

**Table 3:** **Effects of the interactive balance training on functional performance**

Parameters	Control group			Intervention group			P value^b^	Effect size^c^
	Baseline n = 15	Post-test n = 15	% change^a^	Baseline n = 15	Post-test n = 15	% change^a^		
Alternate step test, sec	19.97 ± 7.43	18.77 ± 5.77	6.0	19.49 ± 6.46	15.78 ± 4.98	19.0	0.037	0.151
Timed-up-and-go, sec	17.97 ± 4.86	18.67 ± 5.28	-3.9	16.55 ± 4.72	14.91 ± 5.41	9.9	0.024	0.174
Gait speed, normal, cm/sec	0.77 ± 0.20	0.80 ± 0.20	3.9	0.83 ± 0.16	0.90 ± 0.18	8.4	0.264	0.048
Gait variability, normal, CV	5.86 ± 3.57	4.98 ± 2.30	15.0	5.82 ± 4.92	4.84 ± 3.50	16.8	0.902	0.001
Gait speed, fast, cm/s	1.04 ± 0.20	0.99 ± 0.23	-4.8	1.07 ± 0.20	1.15 ± 0.26	7.5	0.010	0.227
Gait variability, fast, CV	5.08 ± 3.29	5.14 ± 3.78	-1.2	5.04 ± 2.61	5.06 ± 2.88	-0.4	0.951	0.000

Data presented as mean ± standard deviation; ^a^positive scores indicate improvement; ^b^P-values from ANCOVA comparing the effect of the intervention on post-test outcome parameters adjusting for baseline values; ^c^Effect size eta squared from ANCOVA; CV, coefficient of variation.

### Variables associated with improvement in balance and functional performance

**Figure 3:**
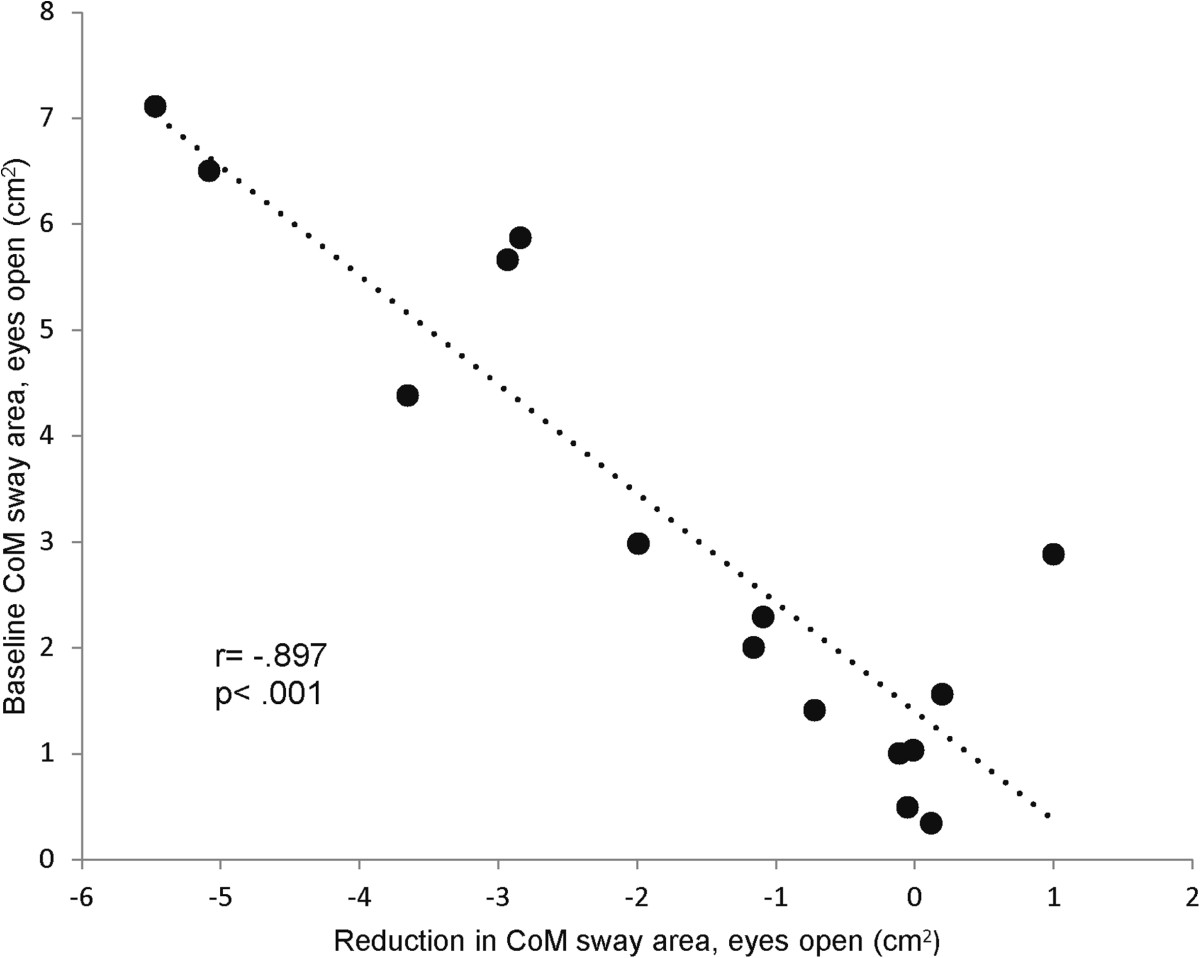
**Association between baseline balance performance and training benefit.** Patients with higher CoM sway at baseline benefited more from the balance training as reflected by a greater reduction in CoM sway after the intervention period.

Low baseline balance performance (higher CoM sway area, EO) was associated with more improvement in the primary study endpoint (pre- to post reduction in CoM sway area, EO: β = -.937, R^2^ = 0.805, p < 0.001) (Figure 3). Other baseline parameters did not significantly predict training response (p = .055 - .994). Three IG participants (20%) did not respond to the exercise intervention (no improvement in the primary study endpoint, CoM sway area EO). Baseline characteristics of non-responders and responders were not significantly different (p = .134-.952).

**Figure 4:**
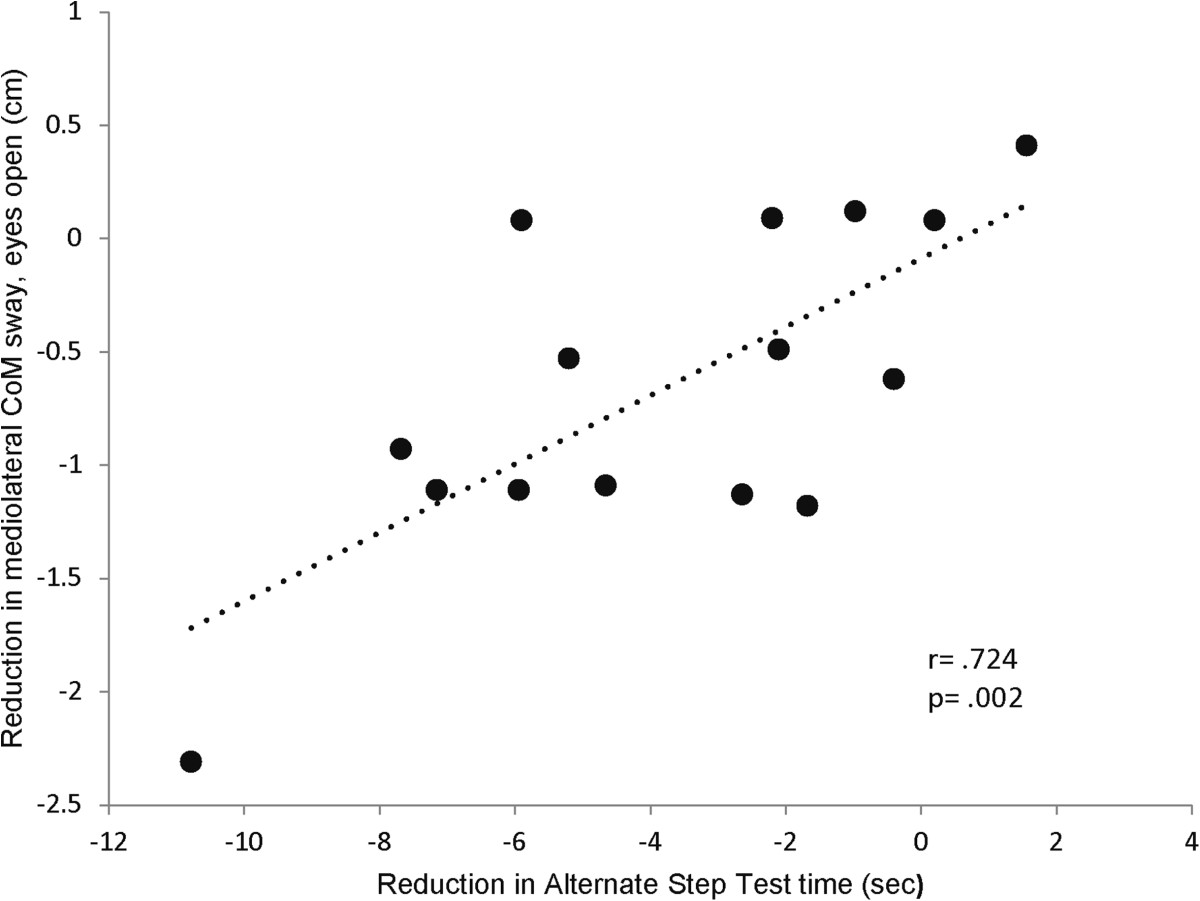
**Association between improvement in standing balance performance and improvement in functional performance.** Improved postural balance during standing (i.e. reduced mediolateral CoM sway) was associated with improved performance in the Alternate Step Test. Negative values in the chart indicate improvement.

Reductions in ML CoM sway and hip sway during balance assessment with EO were significantly associated with reductions in AST (r = .615-.724, p = .002-.015) (Figure 4). Reductions in CoM sway area and AP CoM sway during balance assessment with EC were significantly associated with improvements in fast gait speed (r = -.546 - -.596, p = .025-.043). Changes in other balance parameters were not significantly associated with changes in functional tests (r = -.474-.407, p = .074-.930).

### User-experience

Table 4 shows the descriptive results of the user experience questionnaire in mean, standard deviation, median, and range. The majority of participants absolutely agreed in having fun while exercising; without experiencing problems or safety concerns (mean score > 3.5). The sensor-feedback helped the majority of participants to learn the exercises (mean score = 3.4). Participants agreed moderately that the form and design of the technology was optimal (mean score = 3.1). Most participants disagreed that exercises were too fast or required balance support (mean score < 0.5). Participants disagreed moderately that movements were difficult to perform (mean score = 1.0).

**Table 4:** **Results of the user experience questionnaire**

Question	Mean	SD	Median	Range
**Q1:** It was fun to use the sensor-based balance exercise technology.	3.53	0.64	4	2-4
**Q2:** Usage of the technology was possible without problems at any time.	4.00	0.00	4	4-4
**Q3:** I never lost my balance while using the exercise technology.	3.60	0.91	4	1-4
**Q4:** The form and design of the technology are optimal for me.	3.07	0.88	3	1-4
**Q5:** I was afraid to tumble or to fall during the exercise.	0.20	0.56	0	0-2
**Q6:** I required balance support while conducting the exercises.	0.47	0.99	0	0-3
**Q7:** Thanks to the sensor-feedback, I could quickly learn all exercises.	3.40	0.82	4	2-4
**Q8:** I feel that the exercises were going too fast for me.	0.20	0.41	0	0-1
**Q9:** Some of the movements were difficult to perform.	1.00	0.93	1	0-3
**Q10:** I felt safe using the exercise technology.	3.80	0.41	4	3-4

Answer categories: 0 = disagree completely; 1 = disagree moderately; 2 = neutral; 3 = agree moderately; 4 = agree absolutely.

## Discussion

Results of this pilot study suggest that the proposed balance training program is effective for improving postural control and functional performance in older adults, as well as fun and safe. To our knowledge, this is the first study in older adults integrating wearable sensors into a balance training program for providing real-time feedback about motion performance during exercise.

It should be noted that this proof-of-concept study did not compare the sensor-based balance training to other conventional or exergaming balance training programs. Therefore, this study cannot prove an added value of the sensor-based feedback. A future study with an active control group is required to evaluate whether the positive effects obtained in this study are related specifically to the new training paradigm. Nevertheless, present results are promising, and represent a first step towards evaluating the proposed sensor-based training approach in the target population.

Increased CoM sway in the ML direction has been repeatedly identified as a predictor for future falls [44, 45] and this specific sway component was substantially reduced in our study. Improvements in ML balance control may be related particularly to obstacle crossing practice. During single leg stance phase of obstacle crossing balance control is challenged in the frontal plane [46]. Repeated practice of obstacle crossing in our study may have improved maintenance of balance in the frontal plane, and in turn may have minimized medial-lateral sway during balance testing. Further, the hip load/unload mechanism is challenged during obstacle crossing [46]. Training of the hip load/unload mechanism may have resulted in an improved ML neuromuscular control during standing, as discussed previously [47].

The presented wearable sensor-based balance training program integrated both static and dynamic balance tasks, and results suggest that both AP and ML sway were improved with EO. In contrast, 4-weeks of Wii platform-based static balance training in older adults did not improve ML balance control [23], which was likely related to the exclusion of dynamic stepping tasks. Stepping is an important component of balance training programs [48], but safety concerns [13] and falls [49] have been reported during stepping tasks on force platforms such as Wii. In contrast, the presented training used virtual obstacles, which cannot cause a trip, thus enhancing user safety.

ML sway, but not AP sway changed with EC, although we trained balance in AP direction during ankle reaching task. Higher training response for ML sway may have been related to the specific ML baseline balance deficit in our fall prone participants, as described in previous studies [44, 45]. In healthy individuals body sway is larger in AP as compared to ML direction, mainly due to the inherent structural mechanism of ankle and hip joints [47, 50]. In contrast our participants with confirmed fall risk had higher baseline sway in ML direction, compared to AP. Due to lower ML balance performance participants may have had a better training response for this balance component. Previous training studies in older adults have described that a lower baseline motor performance is a predictor for better training response [51, 52].

Our results are in line with a previous laboratory study which included both static and dynamic balance tasks and reported improved standing balance with EO and EC after 4-weeks visual feedback-based training on a force platform in frail older women [31]. However, this previous study used extensive equipment (Good Balance posture training system, Metitur, TX), which cannot easily be translated into a community or home setting. Therefore, it has been suggested to use wearable systems for implementing interactive balance training programs in geriatric practice [7]. Results of this study suggest that wearable sensor systems may help to translate laboratory-based balance training regimes into community settings, although this needs to be validated in a larger study directly comparing the proposed system with other exercise interventions.

We also explored whether the training affects ankle-hip postural coordination strategy (RCI). The reduction in RCI value found in our study may indicate that ankle-hip postural coordination improved in AP direction, although changes were non-significant (p = .051), and a larger study is required to verify these training effects. Comparison of baseline RCI data of our participants (0.57 ± 0.17 and 0.88 ± 0.10 for AP and ML) with healthy young control subjects (0.70 ± 0.01 and 0.80 ± 0.05) of a previous study [21] suggest that our participants had no impairment in postural coordination in the AP direction, but potentially in the ML direction. However, the lack of validated normative RCI values limits direct comparison of our study participants with those in the general population. It remains unclear whether the (non-significant) changes in AP postural coordination observed in our study are clinically meaningful. Future studies using the present training may select older adults with a specific impairment in postural coordination (e.g. due to a somatosensory impairment caused by a peripheral neuropathy [21, 53]) and link potential training-related changes in RCI to other clinically relevant outcomes such as falls.

IG participants showed improved functional performances after the intervention as measured by gait, TUG and AST. The AST is a complex functional task measuring lateral stability, strength, and movement speed [38]. Our data show that improved AST was strongly associated with improved ML standing balance, which may suggest that gain in postural control resulted in improved functional performance.

Improvements in fast gait speed (0.08 m/sec) represent a clinically meaningful change [54]. The significant association between reduced CoM sway EC and improved gait speed may suggest that gain in balance could be transferred to walking. Further, single leg stance practice during obstacle crossing may have improved gait speed in our participants. Previous studies in older adults have identified a significant correlation between single leg stance time and gait speed [55]. Results are in line with previous exergame studies which reported improvements in gait performance as well as in TUG [6, 56].

Participants with lower baseline balance performance had a better training response. These findings suggest that the most impaired participants reaped the most benefit. Results are in accordance with earlier studies indicating that participants with the lowest performance benefit most from exercise interventions [51, 52].

The positive user experience obtained in this study suggests that this intervention is feasible in the target group and meets important requirements of a home training program, including safety and fun to use. Gerling et al. used the same user experience questionnaire for evaluating the Wii Fit platform in nursing home residents [11]. While these authors found comparable results for fun to use (mean 3.57; our study 3.53), Wii users expressed higher fear of falling (mean 3.14; our study 0.20) and less help from the technology/biofeedback for conducting the exercises (mean 1.29; our study 3.40). These findings suggest that the presented wearable-sensor system has a higher feasibility compared to platform-based exergame systems with better perceived support through sensor-feedback in mastering the exercises. Also, the simplistic design of the graphical user interface used in our study may have accounted for reduced visual and cognitive abilities, and allowed users to focus on the exercise tasks instead of being distracted by complex animations or other graphical effects, as reported for commercial systems [11].

### Limitations and future research

This study has a number of limitations. The effectiveness of the sensor-based balance training was not evaluated against other training programs. It is possible that any form of physical activity may have increased motor performance in our sample of functionally impaired older adults, although results suggest that the balance training induced specific improvements in postural control. Although we observed substantial improvements in balance and functional performance immediately after the intervention, the ideal dosing of the intervention, and sustainability of training effects, remains unclear. Similar to previous proof-of-concept balance training studies [23] the intervention period in this study was short and the frequency of training low. More practice may optimize motor learning and improve retention of benefits [57]. A double-blinded RCT with extended training period, active control groups (i.e. other kinds of attention, conventional balance training, commercial exergames), and long-term follow-up to assess the sustainability of training effects and the effect on the incidence of falls is required to evaluate the true potential of the presented training. Further, potential transfer effects related to the interactive balance training (i.e. cognitive, visuoperceptual) should be assessed in future studies.

We provided assistance to the participant in terms of putting on/off the sensor straps and setting up the software. Thus, results of the user experience need to be interpreted with caution and user experience needs to be evaluated in an unsupervised setting. Assistance in this study was required because we used a prototype of the balance training technology that was not yet designed for fully unsupervised training. Based on the present results we are currently developing a technology for autonomous usage by older adults including Bluetooth sensors, user-friendly computer interface, and automated adjustment of task difficulty for the purpose of unsupervised home training. Although, an exact cost calculation for such system has not been performed, estimates suggest a cost less than $200. In addition, it may be more practical for subjects to exercise in front of the TV screen, instead of in front of a computer screen. We are planning to develop an HDMI dongle that can be plugged directly into any modern TV, allowing exercise in front of a TV. The HDMI dongle is based on an off-the-shelf component with the balance training software preloaded on it and may cost less than $100. It is expected that with time these costs would decrease with economies of scale and outsourcing of components.

## Conclusions

The presented pilot study is an initial step towards evaluating a new balance training system, which has potential for integration in commercial wearable technology applications designed for rehabilitation of frail older adults at risk of falling [5]. Current findings may help to inform tailored interventions integrating wearable sensors for interactive balance training in a home environment. Future studies need to evaluate the added value of the proposed training paradigm compared to existing balance training programs.

## Authors’ original submitted files for images

Below are the links to the authors’ original submitted files for images. Authors’ original file for figure 1 Authors’ original file for figure 2 Authors’ original file for figure 3 Authors’ original file for figure 4

## Abbreviations

ANCOVA: Analysis of covariance
AP: Anterior-posterior
AST: Alternate Step test
CG: Control group
CoM: Center of mass
EC: Eyes closed
EO: Eyes open
IG: Intervention group
ML: Medial-lateral
MMSE: Mini-Mental State Examination
RCI: Reciprocal compensatory index
RCT: Randomized controlled trial
TUG: Timed up and go test.
