# Supplementary figures and images for: Interactive balance training integrating sensor-based visual feedback of movement performance: a pilot study in older adults
Source: J Neuroeng Rehabil. 2014 Dec 13;11:164. doi: 10.1186/1743-0003-11-164 (PMC4290812; doi:10.1186/1743-0003-11-164)

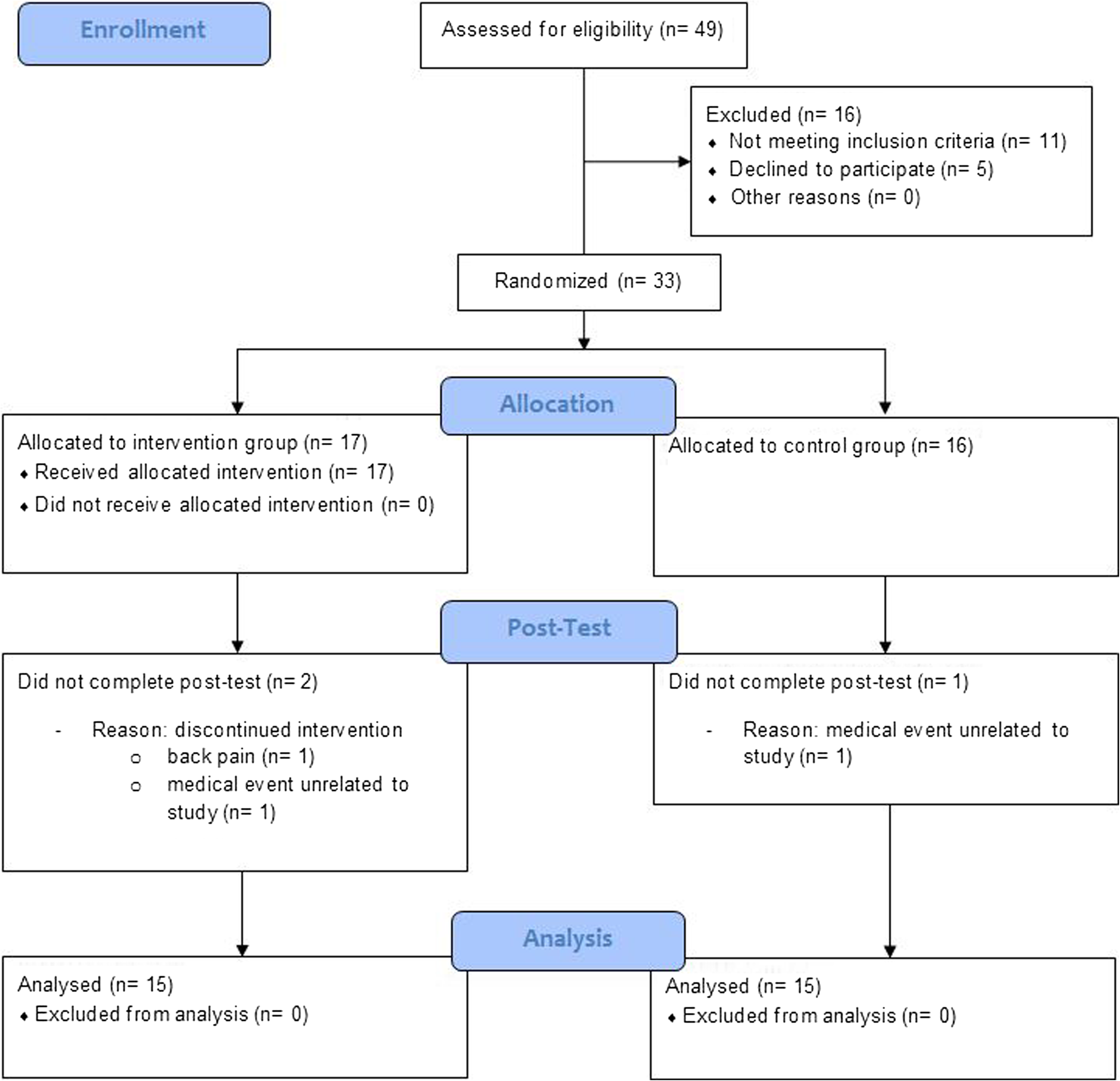

Supplement: Supplementary file 1 — Authors’ original file for figure 1 [file 12984_2014_691_MOESM1_ESM.tif]

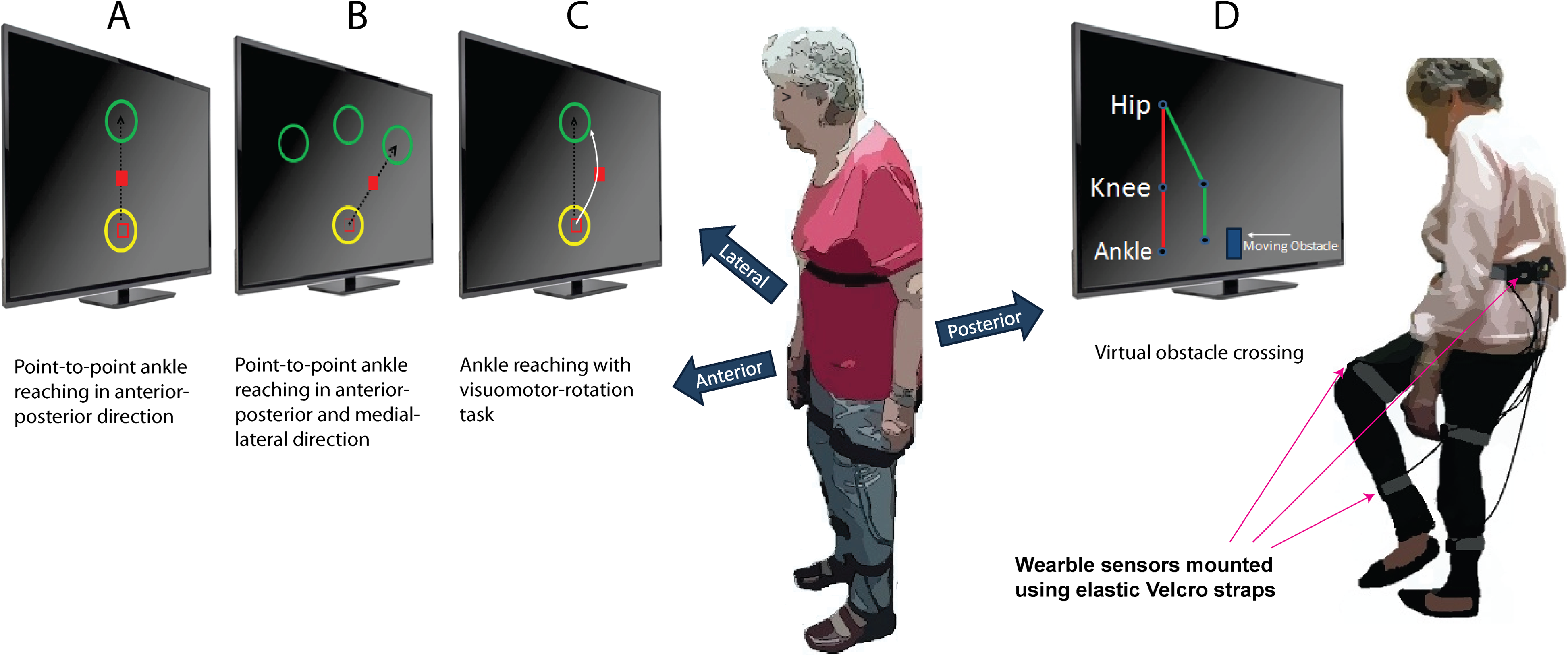

Supplement: Supplementary file 2 — Authors’ original file for figure 2 [file 12984_2014_691_MOESM2_ESM.tif]

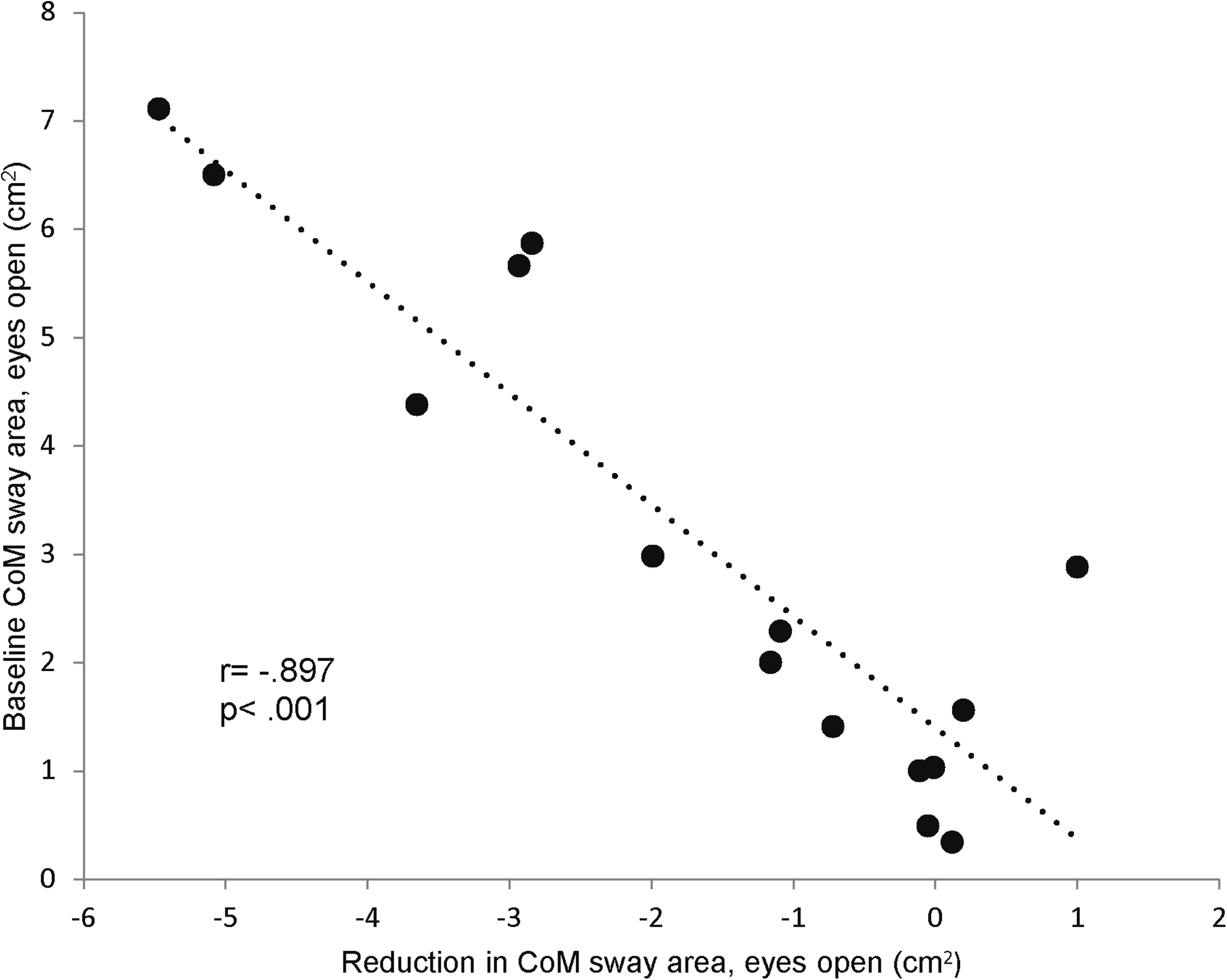

Supplement: Supplementary file 3 — Authors’ original file for figure 3 [file 12984_2014_691_MOESM3_ESM.tif]

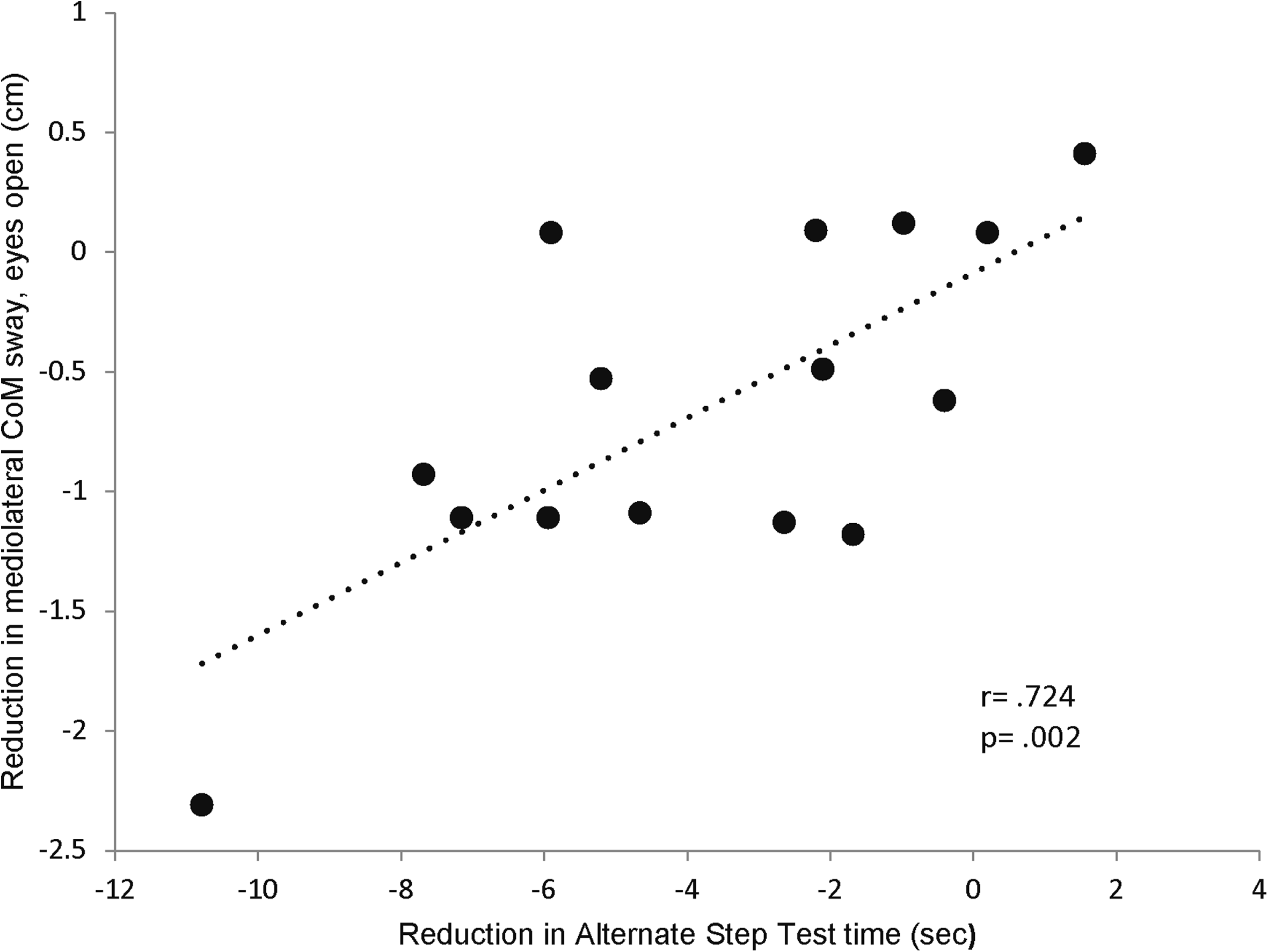

Supplement: Supplementary file 4 — Authors’ original file for figure 4 [file 12984_2014_691_MOESM4_ESM.tif]
